# Scale Structure and Reflectance Activity of *Metapocyrtus apoensis* with Notes on Its Distribution in Mindanao, the Philippines

**DOI:** 10.21315/tlsr2021.32.2.8

**Published:** 2021-06-29

**Authors:** Romeo R. Patano, Joriegae M. Abales, Erika J. Bajuyo, Ara Belle R. Magtulis, Rainear A. Mendez, Rhesa T. Hinampas

**Affiliations:** 1 Center for Biodiversity Research and Extension in Mindanao, Central Mindanao University, Musuan, Maramag, Bukidnon 8710, Philippines; 2 Central Mindanao University Laboratory High School, Musuan, Maramag, Bukidnon 8710, Philippines; 3 Graduate Studies, Department of Biology, College of Arts and Sciences, Central Mindanao University, Musuan, Maramag, Bukidnon 8710, Philippines

**Keywords:** Dipterocarp Forest, Elytra, Endemic, *Metapocyrtus*, Nipple-Like Structure

## Abstract

Scale structure and reflectance activity of a Mindanao endemic weevil from the genus *Metapocyrtus* has been studied for the first time. Specimens of *Metapocyrtus apoensis* Schultze, 1925 were collected through opportunistic sampling in Mount Calayo, Musuan, Mindanao, Philippines last February 2020. A total of three individuals of the species were collected all in lower dipterocarp forest with elevation of 500 masl–600 masl. Three specimens were then examined under Scanning Electron Microscopy with Energy Dispersive X-Ray (SEM_EDX) to analyse its scale structures and reflectance activity. The study provides new locality record of the Mindanao endemic species first in Bukidnon region and an updated distribution in Mindanao based on recent published articles and museum collections. The species inhabits wide ranges of habitat types that greatly differ in elevation and vegetation. Examination of scale’s structure through SEM revealed that *M. apoensis* scales are 50 μm–70 μm in diameter which are almost circular in shape, slightly convex with rough like surface which is termed as non-ordered nipple-like structure. The scales’ shape and surface structure clearly differ from other genera of curculionids based on published articles. Analysis of the particles on the weevil’s elytra done by EDX reveals several elements that contribute to its iridescence. Major elements such as carbon (42.3%), oxygen (27.7%) and nitrogen (15.1%) come in relatively high atomic concentrations. Microspectrometer revealed a peak reflectance wavelength of about 569.7 nm. This explains the yellow-green iridescence observed on the dorsal side of the weevil. The concentration of the scale in pits serves for protection, intraspecific recognition and camouflage. Despite of widespread distribution and high abundance of this species in Mindanao, anthropogenic disturbances such as agricultural activities are on-going which extend towards their microhabitat. Monitoring to its population is recommended as the species is restricted only in Mindanao.

**Highlights**

- New locality records of the Mindanao endemic species, *Metapocyrtus apoensis* Schultze, 1925, specifically in Bukidnon region.
- Scales of *Metapocyrtus apoensis* Schultze, 1925 are 50 μm–70 μm in diameter which are almost circular in shape, slightly convex with rough like surface which is termed as non-ordered nipple-like structure.
- The species scales revealed a peak reflectance wavelength of about 569.7 nm. This explains the yellow-green iridescence observed on the dorsal side of the weevil.

## INTRODUCTION

The Philippines, one of the mega diverse countries (Mittermeier *et al*. 1998), is inhabited by interesting group of beetles called jewel weevils (Coleoptera: Curculionidae). They are called jewel weevils due to their clear pattern and structure of its colourful scales which can be mostly noticed on its elytra termed as photonic crystals (Biro *et al*. 2003; Yablonovitch 1987). These crystals are the most complex structures in living organisms which create colours by splitting the white light in its components (Buechi 2018). Biophotonic crystal on weevils’ scales specifically from the genera *Myllocerus*, *Phyllobius*, *Chlorophanus*, *Pachyrhynchus* and *Entimus* were already been studied by experts abroad in which it aims to determine their structure, sexual dimorphism and camouflage capability (George *et al*. 2015; Tamanis *et al*. 2012; Tamanis *et al*. 2014; Wilts *et al*. 2012a; Wilts *et al*. 2012b). Studying these characteristics is important to understand the components and structure responsible to their colours. These studies link the perspective of biology and physical sciences. Currently, there had been no studies conducted on Mindanao endemic weevils specifically from the genus *Metapocyrtus* when it comes to their scale structure and reflectance activity.

At present, there had been a total 51,000 species of weevils in 4,600 genera worldwide, in which Philippines contributes more than 400 species (Oberprieler *et al*. 2007). Almost 90% of these species are endemic in the archipelago (Ballentes *et al*. 2006). One of its known genera is the *Metapocyrtus*. Recent discoveries on new species of Philippine weevils from the genera *Metapocyrtus* and *Pachyrhynchus* were conducted by Filipino taxonomists and systematists collaborated with taxonomists and entomologists from other countries (Yoshitake 2012a; 2012b; Bollino & Sandel 2015; Rukmane & Barsevskis 2016; Rukmane 2016; Cabras & Rukmane 2016, Cabras *et al*. 2019; Cabras & Medina 2018). Currently, there had been a total of 200 species of *Metapocyrtus* were recorded in the Philippines (Yap & Gapud 2007). One of the known endemic species in Mindanao is *Metapocrytus apoensis* Schultze, 1925. This species was lately recorded from Davao region specifically in Marilog Forest Reserve and Mount Apo (Cabras *et al*. 2016; Mohagan *et al*. 2018).

Thus, this study aimed to update the distribution of *Metapocyrtus apoensis* Schultze, 1925 in Mindanao and provide baseline information on its scale structure and reflectance activity first from the genus through conducting different examinations and activity test under scanning electron microscopy (SEM).

## MATERIALS AND METHODS

### Sampling Sites

**Figure 1:**
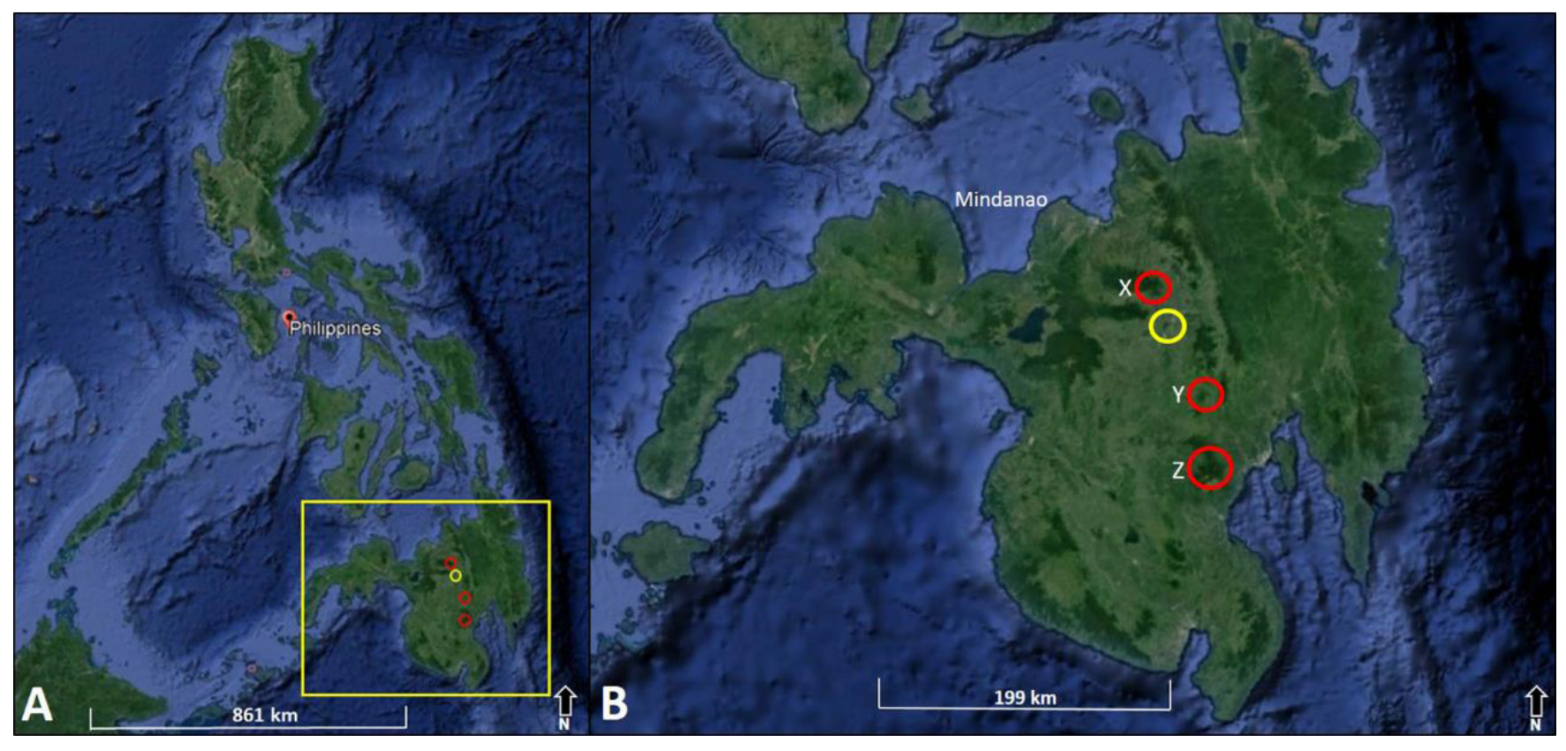
Map of the Philippines (A) and Mindanao (B) showing the location of Mount Calayo, Musuan (yellow circle), Maramag, Bukidnon where the specimens of *Metapocyrtus apoensis* Schultze, 1925 were collected and other known localities of the species (red circles) based on published papers and museum collections. **(X)** Mount Kitanglad Range, Kaatuan, Lantapan, Bukidnon (08°06′29.54″N, 124°56′0.61″E; February 2019; 1,500 masl–1,700 masl); **(Y)** Baganihan, Marilog Forest Reserve, Marilog District, Davao City (07°27′13.74″N, 125°15′1.12″E; February 2018; 1,220 masl–1,240 masl); **(Z)** Mount Apo, Natural Park, Davao City (May–December 2015; 07°0.21′15.05″N, 125°19′77.92″E; 800 masl–1,000 masl). (*Source*: ©2020 Google Earth, image © 2018 CNES/Airbus)

Mount Calayo, Musuan, Maramag, Bukidnon was surveyed last February 2020 (Fig. 1). This area was surveyed due to its intact forest and accessibility. This mountain ecosystem is one of the Long-Term Ecological Research (LTER) sites located at central Mindanao with an elevation of 400 masl–750 masl (07°52′14.16″N, 125°04′08.75″E). This area is a known landmark in the locality of Bukidnon lying beside the main road. The area can be considered as evergreen forest surrounded by agroforest ecosystem due to shifting cultivation of farm crops such as *Zea mays* L., *Cocos nucifera* L. and *Theobroma cacao* L. by the local people.

The area is dominated by flowering plants primarily from the family Euphorbiaceae, Annonaceae and Fabaceae. Understory plants are dominated by aroids (*Alocasia* sp.), gingers (*Etlingera* sp.) and ferns (*Christella* sp.). Water systems were also observed in the area such as creeks, canals and swamps. The forest floor is mostly covered with leaf litters of tall trees.

### Collection of Weevils

The standard belt-transect sampling method with combination of opportunistic sampling was employed in the study (Heyer *et al*. 1994). The specimens of *Metapocyrtus apoensis* was encountered along the transect covering 10 m by 5 m on both sides. Specimens were collected through handpicking whenever encountered during the diurnal (0700–1500) sampling. Collected specimens were killed in jars with ethyl acetate then preserved in silica gel and brought to the laboratory, air-dried and photographed. Utmost three individuals of the species were collected in accordance to the Wildlife Gratuitous Permit No. R10 2020-04 issued by the Department of Environment and Natural Resources (DENR).

### Stereomicroscopy, Identification, Morphometrics and Descriptions

A typical specimen of *M. apoensis* was examined under a stereomicroscope and photographed using a Canon camera. Following measurements were then taken in accordance with Schultze (1923) and Yoshitake (2012): Body length (from the apical margin of pronotum to the apex of the elytra), elytral length (from the level of the basal margins to the apex of the elytra), elytral width (maximum width across elytra), pronotal length (from the base to apex along the midline), pronotal width (maximum width across pronotum), rostrum length and rostrum width (maximum width of the rostrum). Published articles, taxonomic keys and monographs of Schultze (1923) and Yap and Gapud (2007) were used as guides for the description and identification of the species. The collected specimens were initially identified (RRPJ) and confirmed by Dr. Analyn Anzano Cabras, an expert of Philippine weevils from University of Mindanao.

### Diagnosis of Biophotonic Crystals and Determination of Nanostructures

Scanning Electron Microscope (SEM) was used to obtain real space information on the structure of the scales while the Energy Dispersive X-Ray (EDX) was used for the elemental analysis of the weevil’s scales. A reflectance analysis was also done using the ultraviolet visible (UV-Vis) spectrophotometer.

## RESULTS AND DISCUSSION

### Diagnosis of the Species

The legs are dominantly chromatic reddish orange in the femur with dense coloured black segments tracing down in its mid-femur and tarsus. The body is black almost covered with green to yellow stripes of scales.

### Material Examined in the Study

Altogether three specimens—one male, two females PHILIPPINES: (1/3) 1♀ Mount Calayo, Musuan, Maramag, Bukidnon 8°27′73.0″N, 125°36′54.6″N, 600 masl, 1 February 2020, R.R. Patano Jr., J.M. Abales, E.J. Bajuyo, A.B.R. Magtulis, and R.T. Hinampas, Central Mindanao University, University Museum, Zoological Section.

### Distribution

Inhabiting tropical mountainous rainforests of Mindanao (the Philippines) at 500 m–1,700 m above sea level: known from Davao region by Mohagan *et al*. (2018, 2020) and Cabras *et al*. (2016) and Bukidnon Region (present study) (Table 1).

### Measurements in Millimeters

Morphological data of the male *Metapocyrtus apoensis* in terms of its length and width were measured. Total body length = 6.6 mm, elytral length = 3 mm, elytral width = 1.9 mm, pronotal length = 1.6 mm, pronotal width = 1.7 mm, rostum length = 1.99 mm and rostum width = 0.88 mm.

### Habitat and Ecology

**Figure 2:**
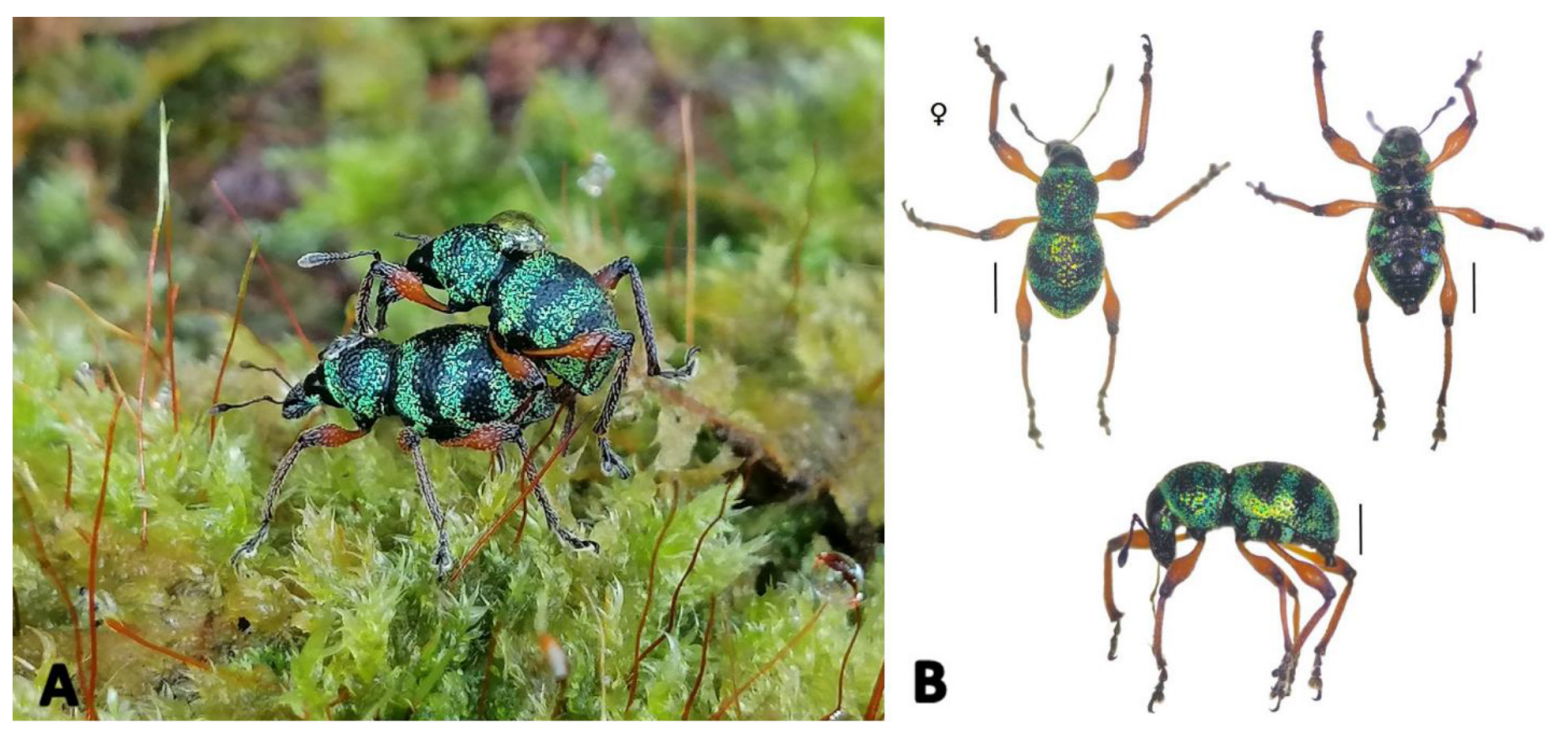
(A) *M. apoensis* Schultze, 1925 on its natural habitat perching and copulating on a shrub covered with mosses in Mount Calayo, Musuan, Mindanao, Philippines. (B) Dorsal, ventral and lateral view. Scale bars = 1 mm

The specimens were mostly observed perching and copulating on shrub plant species (*Costus* sp.), mosses and ferns (*Christella* sp.) at the lower evergreen forest of Mount Calayo, Musuan, Maramag, Bukidnon and the distribution of the species are secluded but rich as per observed during the collection (Fig. 2). It is distinctive and rare to find *Metapocyrtus* species in Mt. Calayo because of its elevation which is not as high compared to the elevation of Marilog Forest Reserve, Mt. Apo, Mt. Kitanglad, and other prominent mountains found in Mindanao wherein this said species inhabit dominantly. This implies that *M. apoensis* belongs to those species of *Metapocyrtus* that are highly adaptive to environmental changes and with no specific food preference which enable them to survive in wide ranges of habitat types especially in lower elevation.

### Diagnosis of Biophotonic Crystals through Ultrastructure of the Scale

**Figure 3:**
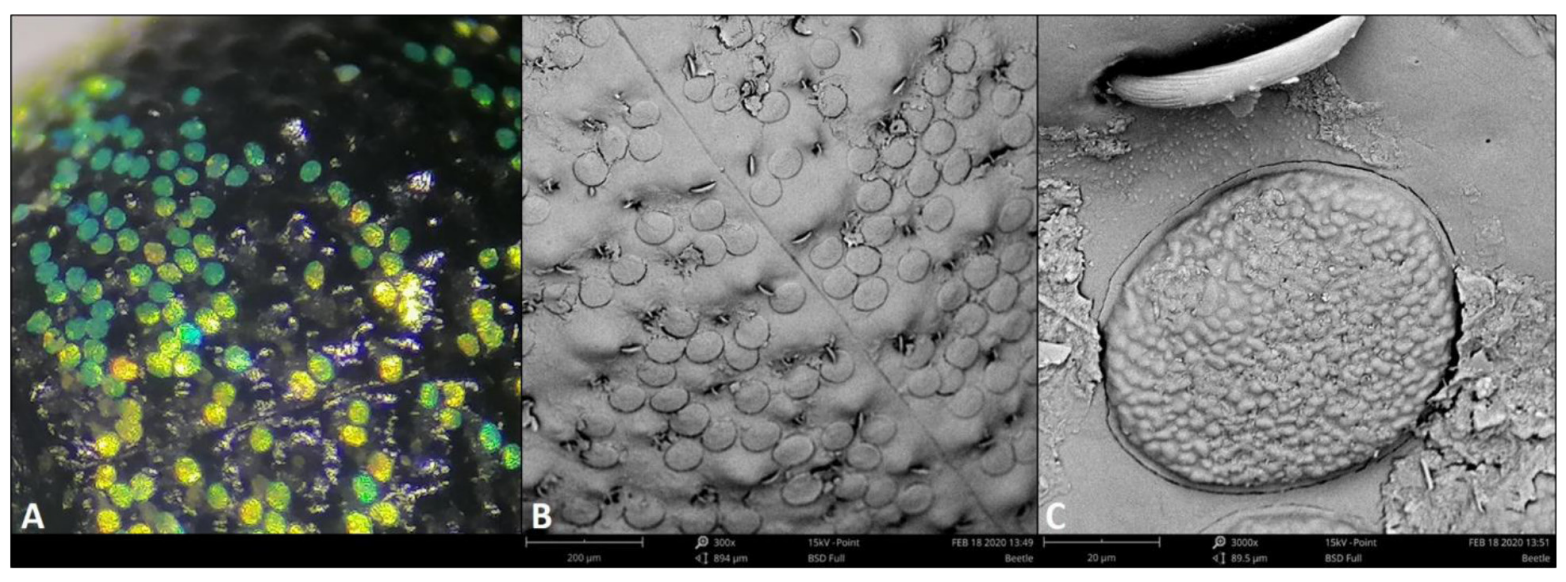
*M. apoensis* Schultze, 1925 elytral scales (A) and its surface structures (B&C) through SEM.

*M. apoensis* has an overall black coloured body with a head and elytra almost covered with dense blue, green and yellow spots (Fig. 3A). The striped horizontal pattern of the colouring is prominent in the dorsal view within the area of the head and elytra with the thorax having less area of coloured spots. The dorsal view shows that the abdomen is mostly dominated by black with striped-pattern spots on both sides. It has a relatively uniform and metallic colour. This colour is derived from scales that are about 50 μm–70 μm in diameter and occur in patches on the top and sides of the beetle’s roughly hemispherical body. The hairs are scattered irregularly throughout the body with total length of 60 μm–70 μm and 6 μm–7 μm total width. Individually, the scales are almost circular to oval in shape, rough and slightly convex parallel with the body (Figs. 3B and 3C). The rough surface of the weevil scale is termed as irregularly nipple-like structure or array. This type of surface structure was only observed in the cornea of butterflies (Stavenga *et al*. 2011). The *M. apoensis* Schultze, 1925 scale’s surface structure differs from other genera of curculionids by having a non-ordered nipple-like surface.

In comparing other scale structures from other genera of curculionids, difference in arrangements, sizes, colours and surface orientations were observed. Weevils from the genera *Phyllobius* and *Chlorophanus* are having scales that are rounded to oblong in shape with corrugated surface which are called as opal structures (Tamanis *et al*. 2012; Tamanis *et al*. 2014). The species *Entimus imperialis* had this jet black elytra with rows of brilliant green spots which are having a slightly corrugated surface (Wilts *et al*. 2012b). They have longer scales compared to *M. apoensis* having length of 100 μm and width of 50 μm. Another species from the genus *Entimus* (*E. cuvieri*) had this cyan-green, turquoise to yellow orange coloured scales which are nearly circular in shape. Although they have almost the same surface orientation with *E. imperialis*, they have much bigger scales having diameter of 140 μm (Wilts *et al*. 2012b). Another weevil species from the genus *Myllocerus* had these plumose scales which are oval to obovate in shape with ridged surface that provides them with white appearance (George *et al*. 2015).

*Pachyrhynchus congestus* is the first species of weevil endemic in the Philippines to undergo photonic examination. This is the other well-known genus of weevils in the archipelago other than *Metapocyrtus*. They have elongated scales with corrugations having 100 μm length, 50 μm width and 5 μm thickness. Multilayered scales were observed which are being covered with discernable scales which give them orange colors in some areas (Wilts *et al*. 2012b).

It can be observed that scales from different genera of curculionids have almost same shapes and sizes but clearly differs in surface structures. *M. apoensis* had this unique oval to almost circular shape scales with nipple-like surface which is first to observed in a weevils’ scale. Moreover, the present study provides the first scale structure from the genus *Metapocyrtus*. Scales structures may differ across siblings of the genus, but size and surface may vary. Providing the first scale structure from the genus is important not just in understanding their photonic activities but adding a potential taxonomic tool by comparing scale structures from other siblings in the future.

### Scanning Electron Microscope (SEM) with Energy Dispersive X-Ray (EDX) Analysis

**Figure 4:**
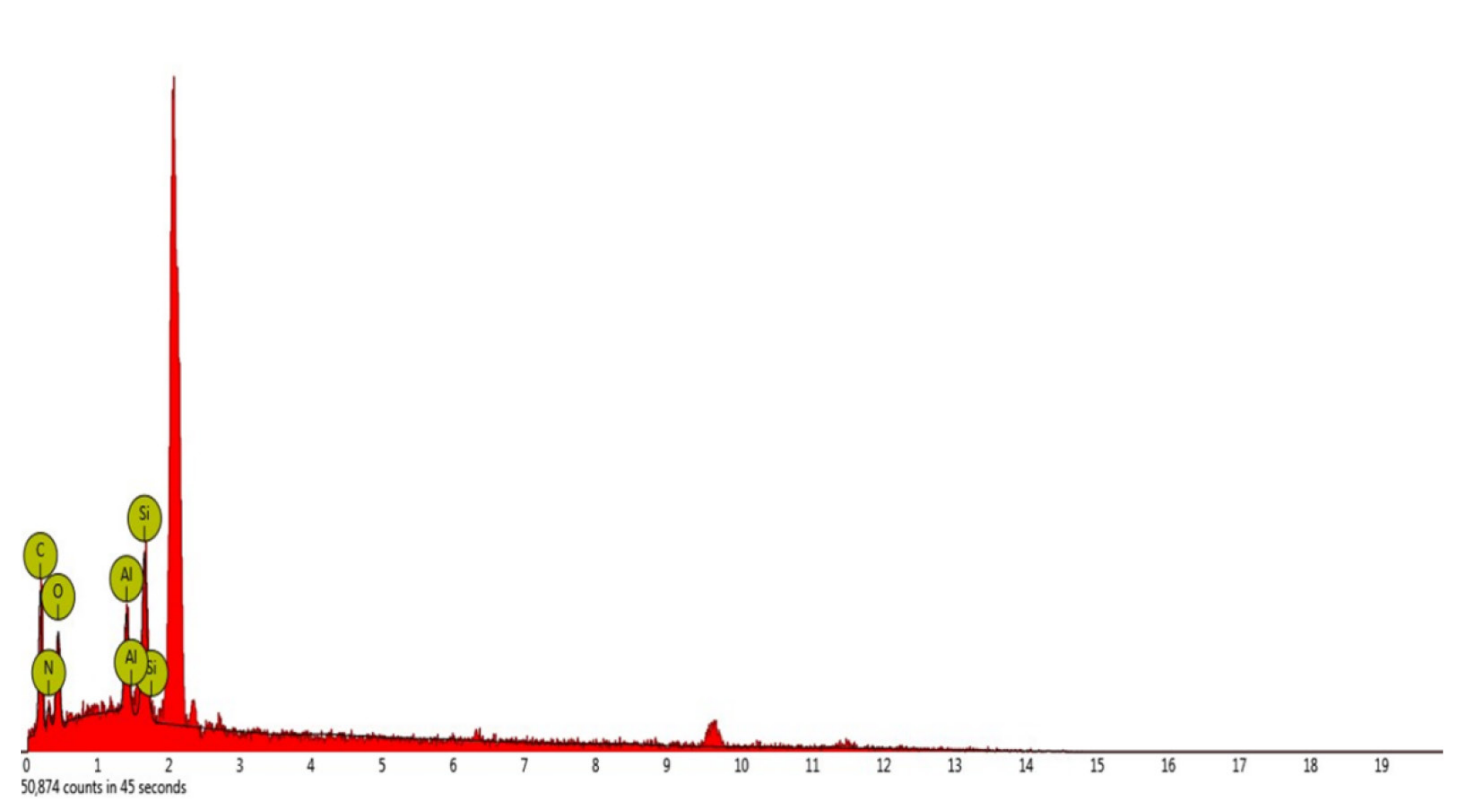
Scanning Electron Microscopy with Energy Dispersive X-Ray (SEM_EDX) result showing major elements on the spot of *M. apoensis* Schultze, 1925 elytra.

Analysis of the particles on the weevil’s elytra done by EDX reveals several elements that contribute to its iridescence. Carbon, oxygen and nitrogen comes in relatively high atomic concentration (Table 2 and Fig. 4). Carbon constitutes 42.3% of atomic concentration with 51.2% weight concentration, followed by oxygen which constitutes 27.7% atomic concentration with 25.2% weight concentration and nitrogen with 15.11% atomic concentration and 15.7% weight concentration. Other elements also detected such as silicon with concentration of 4.85% and weight concentration of 9.35%, and lastly, aluminum with concentration of 3% and weight concentration of 5.57.

### Reflectance Spectra Measured through Microspectrophotometer from Weevil’s Scale

**Figure 5:**
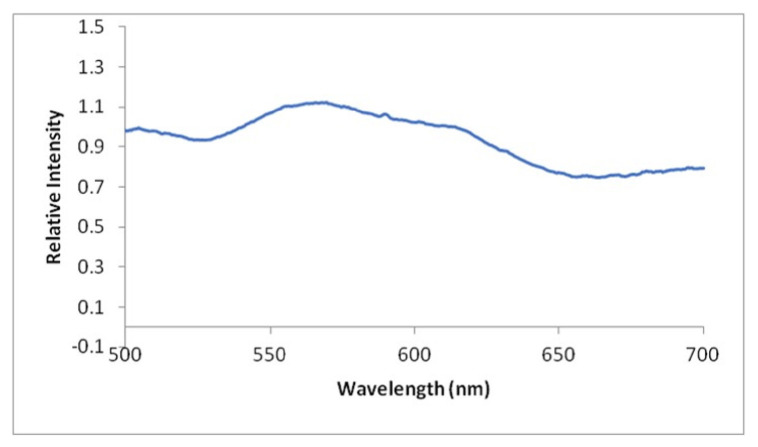
Graph showing the reflectance activity of the dorsal scales of *M. apoensis* Schultze, 1925.

To quantify the observed scale colours, the reflectance spectra of the weevil’s scale were measured with a spectrometer. When analysed using an ultraviolet-visible spectrometer (UV-Vis spectrometer), with normal incidence illumination at 90° with respect to the surface, peak reflectance occurs at a wavelength of 569.7 nm (Fig. 5). The maximum spectrum denotes the primary colour of the species. This explains the yellow-green iridescence observed on the dorsal side of the weevil. The rest of the visible spectrum is slightly weakly reflected, which only very slightly desaturates the yellow-green hue of the insect. Measurement of the integral reflectance of the scale assembly in elytral pits yielded spectra matching the spectrum of a green, foliaceous background, the weevil’s natural habitat (Wilts *et al*. 2012a). This suggests that the scale set is optimised for camouflage for distant predators. However, the concentration of the glittering scales in distinct pits causes a spotted patterning for observers at close range. The bright patterning may allow ready recognition for nearby nonspecific. These capabilities of scales were first observed from the genus *Entimus* (Wilts *et al*. 2012b).

## CONCLUSION AND RECOMMENDATION

The study provides updated distribution of *M. apoensis* Schultze, 1925 in Mindanao with new locality records in Bukidnon regions specifically in Mount Calayo, Musuan and Mount Kitanglad, Lantapan. Moreover, the habitat and ecology of the species were observed in which it inhabits wide ranges of habitat types that greatly differs in elevation and vegetation. Using spectrophotometry, benchmark information of this Mindanao endemic weevil found out that its scales are 50 μm–70 μm in diameter which is almost circular in shape. An irregular nipple-like scale surface is first observed in a weevils’ scale specifically from the genus *Metapocyrtus*. Major element components of the scale include Carbon, oxygen, nitrogen, silicon and aluminum. Reflectance activity revealed a wavelength of 569.7 nm which explains the yellow-green iridescence on the species’ dorsum which primarily functions for camouflage against its prey. The study provides the reflective mechanism first for the genus which is already a good start in photonic research in the Philippines specifically on the rare and endemic weevil species. The study highly recommends testing other Philippine endemic weevils to differentiate scale structures and reflectance activities across species which can be a potential tool in understanding the photonic crystals and their taxonomy.

## Figures and Tables

**Table 1:** Localities in from which the *M. apoensis* Schultze, 1925 is known, together with the information on coordinates, elevation, date of research and references each.

Locality	Coordinates	Elevation	Date	Reference
Mount Apo, Natural Park, Davao City	07°0.21′15.05″N, 125°19′77.92″E	800 masl–1,000 masl	May–December 2015	Cabras *et al*. (2016)
Baganihan, Marilog Forest Reserve, Marilog District, Davao City	07°27′13.74″N, 125°15′1.12″E	1,220 masl–1,240 masl	February 2018	Mohagan *et al*. (2018, 2020)
Mount Kitanglad Range, Kaatuan, Lantapan, Bukidnon	08°06′29.54″N, 124°56′0.61″E	1,500 masl–1,700 masl	February 2019	Museum collection
Mount Calayo, Musuan, Maramag, Bukidnon	07°52′14.16″N, 125°04′08.75″E	500 masl–600 masl	February 2020	Present study

**Table 2:** Element composition on *M. apoensis* Schultze, 1925 elytral spot using EDX.

Element name	Atomic concentration (%)	Weight concentration (%)
Carbon (C)	51.21	42.25
Oxygen (O)	25.23	27.73
Nitrogen (N)	15.70	15.11
Silicon (Si)	4.85	9.35
Aluminum (Al)	3.0	5.57
